# The Beat

**Published:** 2008-10

**Authors:** Erin E. Dooley

## Toy Safety Bill Signed

In 2007, millions of toys were pulled from U.S. shelves due to high levels of lead. Legislation passed 14 August 2008 sharply limits the lead allowed in children’s products to 100 ppm, bans the use of phthalates (3 types will be permanently banned, while 3 others are suspended pending further study), and significantly increases funding and staffing for the Consumer Product Safety Commission. The law also mandates testing and safety certifications for products marketed for children ages 12 and under and requires foreign manufacturers to comply with U.S. toy safety standards.

## Arctic Split

**Figure:**
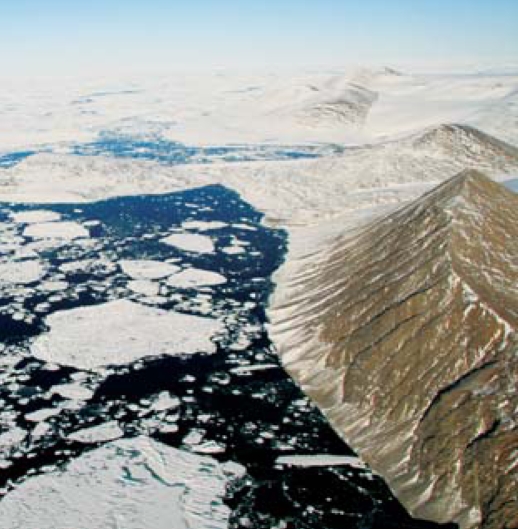
Fragments of the Markham Ice Shelf (left) after splitting off Ellesmere Island (right)

Researchers announced in September 2008 that Canada’s Markham Ice Shelf and two other large tongues of ice had split off Ellesmere Island into the Arctic Ocean, a rate of calving the researchers say highlights the rapidity of climate-related changes occurring in the Arctic. This year, the area of floating Arctic sea ice was at its second lowest point since measurements began 30 years ago. Ice cover reflects radiation from the sun back into space, creating a cooling effect on the Earth; as the area of ice diminishes, dark seawater and ice-free land absorb radiation and could possibly accelerate warming of the Earth. Loss of ice shelves also affects unique local ecosystems that depend on such ice.

## Wastewater for Urban Gardens?

Amid rising scarcity of food and clean water alike, urban farms are an important source of fresh produce in developing countries. But the untreated wastewater used to irrigate many of these farm plots can contain heavy metals and raw sewage, putting the health of potentially hundreds of millions of people at risk. A survey of 53 cities in developing countries released in August 2008 by the International Water Management Institute finds the use of untreated wastewater on urban gardens is “widespread and practically inevitable” due to water scarcity, transport, and monitoring issues. In the study area alone, more than 1 million urban farmers provide produce for 4.5 million people.

## Body Shops Go Green

Automobile body shop owners investing in new water-based paint systems and dust removal technologies are seeing such benefits as lower operating costs, improved air quality, and better worker health. The new paints, which consume fewer solvents and energy as well as speed up the painting process, will be mandatory in California at the end of 2008. In January 2008, the U.S. EPA announced new rules requiring body shops to minimize emissions of paint-stripping chemicals and improve ventilation of workspaces and application equipment, requirements that are expected to reduce pollutant emissions by almost 7,000 tons annually.

**Figure:**
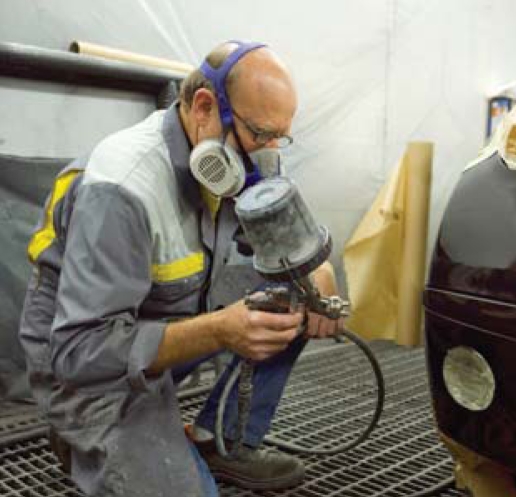
Going clean could pay off for body shops

## Concrete Data on Hg Emissions

Cement manufacturing often uses fuels and raw materials with high mercury content, which is vaporized and emitted through smokestacks. In July 2008, the advocacy groups Earthjustice and the Environmental Integrity Project released a report examining new EPA data that show unregulated mercury emissions from cement kilns to be nearly twice as high as the agency estimated in 2006—almost 23,000 pounds of mercury released annually from more than 150 plants. In some cases cement plants are among the worst mercury polluters in states, surpassing coal-fired power plants. The report calls on the EPA to enact a mercury standard for kilns.

## High-Flying Energy Source

Research teams around the world are working to tap the energy potential of high-altitude winds, a more abundant and reliable energy source than the ground-level winds upon which conventional turbines rely. In an August 2008 proof of concept experiment at the Delft University of Technology, researchers flying a 10-m^2^ kite successfully generated enough electricity to power 10 homes; they believe a full-scale version of the “Laddermill” rig could power some 100,000 homes. Northwestern European countries are thought to be best positioned geographically to take advantage of the high-speed jet stream.

**Figure:**
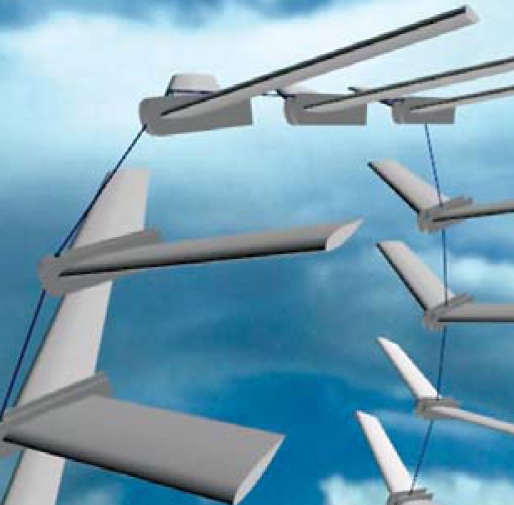
Artist’s rendering of the Laddermill

